# Supplementary material for: Gap junctions set the speed and nucleation rate of stage I retinal waves
Source: PLoS Comput Biol. 2019 Apr 29;15(4):e1006355. doi: 10.1371/journal.pcbi.1006355 (PMC6508742; doi:10.1371/journal.pcbi.1006355)
Supplement: S1 Text — (PDF) [file pcbi.1006355.s001.pdf]

S1 Text

In the main text we used an analytical approximation of the mean membrane potential during a burst to derive an estimate for the propagation speed of GJ mediated waves. In the following, we provide all details necessary to arrive at the equations that we discussed above.

Approximate Solution of the Voltage Equation with Constant Parameters

All approximations are based on solving the time dependence of the membrane potential, Eq. (1), which becomes analytically feasible only by decoupling the system Eq. (1-3). We are specifically interested in the propagation mechanism, and thus in the time the voltage needs to go from the vicinity of the resting potential to the peak potential that marks the occurrence of the first spike. Consequently, the reset mechanism Eq. (3) can be discarded. Until this first spike time, the gating variable u can roughly be regarded as constant, cf. the phase space trajectory displayed in Fig. 1(b). Accordingly, we replace u_i in Eq. (1) by the constant $u_\mathrm{r} = -19.2$ mV., i.e. the resting value of the gating variable. The resulting expression is still a system of coupled differential equations, due to the interaction with neighboring neurons in the network, that is given by the GJ current Eq. (5). To circumvent this problem we simply replace the membrane potential of a bursting neighboring neuron by the constant value $\bar{V}_\mathrm{b}$, an estimate of the temporal average of a neuron's voltage during the burst, which is described in detail below. The voltage of the silent neighbors is replaced by the resting potential. For the discussion of the propagation mechanism these replacements are fairly reasonable, because the propagation process can be thought of as a certain number of synchronously bursting neurons (typically, one or two) exciting a connected neighbor with additional quiescent neighbors.

Under these conditions, the dynamics Eq. (1) can generally be recast into the form

$$\frac{dV}{dt} = \frac{a}{\tau_\mathrm{V}}(V - V_1)(V - V_2). \tag{A}$$

This equation can be solved by separating the variables and integrating. It is however necessary to distinguish the two cases of V_1 and V_2 being real valued (corresponding to the situation with a stable resting potential) or complex valued (unstable situation). We will refer to real valued (V_1, V_2) as $(V_\mathrm{r}, V_\mathrm{c})$ with $V_\mathrm{r} < V_\mathrm{c}$. Real values are e.g. obtained for the case of an isolated neuron, or equivalently $G = 0$, and are related to our original parameters as follows

$$\begin{aligned} V_\mathrm{r} &= \frac{V_\mathrm{rest} + V_\mathrm{crit}}{2} - \sqrt{\frac{(V_\mathrm{crit} - V_\mathrm{rest})^2}{4} + \frac{u_\mathrm{r}}{a}} \\ V_\mathrm{c} &= \frac{V_\mathrm{rest} + V_\mathrm{crit}}{2} + \sqrt{\frac{(V_\mathrm{crit} - V_\mathrm{rest})^2}{4} + \frac{u_\mathrm{r}}{a}} \end{aligned} \tag{B}$$

leading to $(V_\mathrm{r}, V_\mathrm{c}) = (-64\text{ mV}, -60\text{ mV})$ for our parameter choice. In this case, the integration of Eq. A yields

$$\begin{aligned} t &= \frac{\tau_\mathrm{V}}{a} \int_{V(t=0)=V_0}^{V(t)} dV' \frac{1}{(V' - V_\mathrm{r})(V' - V_\mathrm{c})} \\ &= \frac{\tau_\mathrm{V}}{a(V_\mathrm{r} - V_\mathrm{c})} \ln\left(\frac{V(t) - V_\mathrm{r}}{V(t) - V_\mathrm{c}} \cdot \frac{V_0 - V_\mathrm{c}}{V_0 - V_\mathrm{r}}\right). \end{aligned} \tag{C}$$

which can be inverted and thus leads to the explicit solution for the voltage trajectory:

$$V(t) = \frac{V_\mathrm{r} - V_\mathrm{c}\frac{V_0 - V_\mathrm{r}}{V_0 - V_\mathrm{c}}\mathrm{e}^{a(V_\mathrm{r}-V_\mathrm{c})t/\tau_\mathrm{V}}}{1 - \frac{V_0 - V_\mathrm{r}}{V_0 - V_\mathrm{c}}\mathrm{e}^{a(V_\mathrm{r}-V_\mathrm{c})t/\tau_\mathrm{V}}}. \tag{D}$$

Turning to the case of complex valued (V_1, V_2), we will refer to them as $(V_\mathrm{m} + i\gamma, V_\mathrm{m} - i\gamma)$. Integration of Eq. A yields

$$\begin{aligned} t &= \frac{\tau_\mathrm{V}}{a}\int_{V_0}^{V(t)} dV' \frac{1}{V(t)^2 - 2V(t)V_\mathrm{m} + V_\mathrm{m}^2 + \gamma^2} \\ &= \frac{\tau_\mathrm{V}}{a\gamma}\left[\arctan\left(\frac{V(t) - V_\mathrm{m}}{\gamma}\right) - \arctan\left(\frac{V_0 - V_\mathrm{m}}{\gamma}\right)\right]. \end{aligned} \tag{E}$$

The explicit expression of V_m and γ depend on the specific setup of the neighbors (see below).

Approximation of the Mean Voltage During a Burst

To analytically understand the propagation of the burst from cell to cell, we approximate the time-dependent driving voltage of the bursting cell by its time average, $\bar{V}_\mathrm{b}$, resulting in the GJ current:

$$RI_\mathrm{gap}(t) \approx G(\bar{V}_b - V(t)). \tag{F}$$

This replacement is justified because during the period of interest (the time needed for the driven cell to generate a spike), the bursting neuron generates at least a few spikes, i.e. changes rapidly compared to the voltage of the driven neuron.

The mean voltage during the burst can be estimated by integrating the voltage over one inter-spike interval T_ISI, for simplicity considered for an isolated cell ($G = 0$) that is started at $V(t=0) = V_\mathrm{reset}$, $u(t=0) = u_\mathrm{r}$,

$$\bar{V}_b = \frac{1}{T_\mathrm{ISI}}\int_0^{T_\mathrm{ISI}} dt V(t), \tag{G}$$

where $T_\mathrm{ISI} = t(V_\mathrm{peak}) - t(V_\mathrm{reset})$. Using Eq. C, we find

$$T_\mathrm{ISI} = \frac{\tau_\mathrm{V}}{a(V_\mathrm{r} - V_\mathrm{c})}\ln\left(\frac{V_\mathrm{peak} - V_\mathrm{r}}{V_\mathrm{peak} - V_\mathrm{c}}\cdot\frac{V_\mathrm{reset} - V_\mathrm{c}}{V_\mathrm{reset} - V_\mathrm{r}}\right). \tag{H}$$

Using Eq. D and Eq. H in Eq. G, we can calculate the integral and further simplify the resulting expression

$$\begin{aligned} \bar{V}_\mathrm{b} &= \frac{1}{T_\mathrm{ISI}}\int_0^{T_\mathrm{ISI}} dt V(t) \\ &= \frac{1}{T_\mathrm{ISI}}\left[V_\mathrm{r} t - \frac{\tau_\mathrm{V}}{a}\ln\left(1 - \frac{V_\mathrm{reset} - V_\mathrm{r}}{V_\mathrm{reset} - V_\mathrm{c}}\mathrm{e}^{a(V_\mathrm{r}-V_\mathrm{c})t/\tau_\mathrm{V}}\right)\right]_0^{T_\mathrm{ISI}} \\ &= V_\mathrm{r} - \frac{\tau_\mathrm{V}}{aT_\mathrm{ISI}}\ln\frac{1 - \frac{V_\mathrm{reset} - V_\mathrm{r}}{V_\mathrm{reset} - V_\mathrm{c}}\mathrm{e}^{a(V_\mathrm{r}-V_\mathrm{c})T_\mathrm{ISI}/\tau_\mathrm{V}}}{1 - \frac{V_\mathrm{reset} - V_\mathrm{r}}{V_\mathrm{reset} - V_\mathrm{c}}} \\ &= \frac{V_\mathrm{r}\ln\frac{V_\mathrm{peak} - V_\mathrm{r}}{V_\mathrm{reset} - V_\mathrm{r}} + V_\mathrm{c}\ln\frac{V_\mathrm{reset} - V_\mathrm{c}}{V_\mathrm{peak} - V_\mathrm{c}}}{\ln\frac{V_\mathrm{peak} - V_\mathrm{r}}{V_\mathrm{reset} - V_\mathrm{r}} + \ln\frac{V_\mathrm{reset} - V_\mathrm{c}}{V_\mathrm{peak} - V_\mathrm{c}}}. \end{aligned} \tag{I}$$

For our standard parameters this gives $\bar{V}_\mathrm{b} \approx -34$ mV ($T_\mathrm{ISI} \approx 73$ msec).

Propagation Speed in the Three-Neurons Approximation

Let us now turn to the situation, where an initially quiescent neuron is driven to a burst due to the GJ current that results from one bursting neighbor in a one-dimensional chain. To this end, we consider the three neurons $i-1$ (bursting), i (to be excited), and $i+1$ (quiescent). Neuron i is brought from the resting potential V_r to the peak potential V_peak in a period $T_\mathrm{ISI} + T_\mathrm{B}$ that approximately consists of one inter-spike interval T_ISI (the time needed by neuron $i-1$ to go from reset to peak potential for the first time) and the BOTD T_B that determines the speed of the wave via

$$v_{1D} \quad = \quad \frac{\ell}{T_\mathrm{B}}. \tag{J}$$

During the period $t \in [-T_\mathrm{ISI}, T_\mathrm{B}]$ (light blue shaded area in Fig. A(b)), we approximate

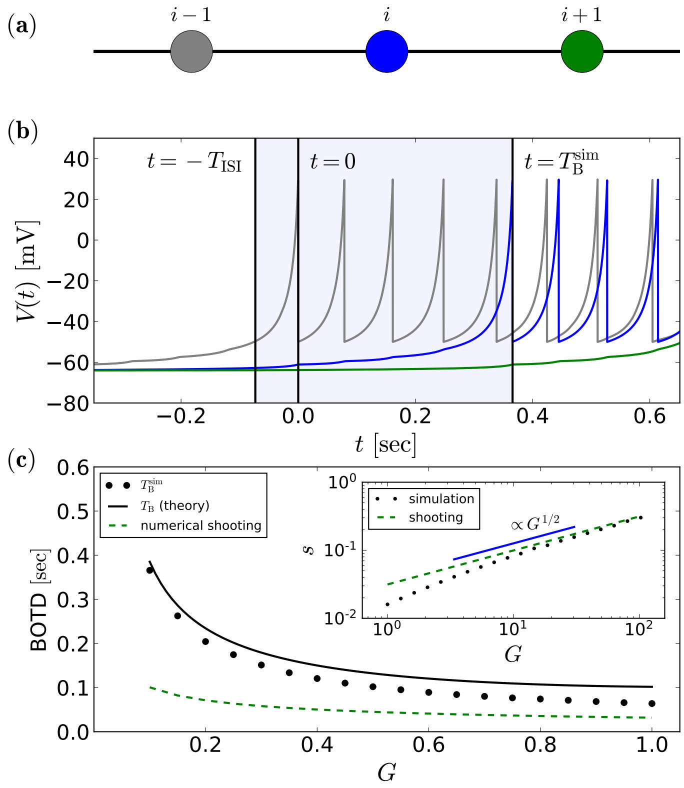

Figure A. Burst propagation and burst onset time difference in the one-dimensional chain. Schematic illustration of the one-dimensional setup **(a)**. Voltage traces of the neurons in **(a)** for $G = 0.1$ shown in **(b)** in respective colors. The shaded area indicates the time period relevant for the excitation of neuron i (blue line) by the GJ current from neuron $i-1$ (gray line). The membrane potential of neuron $i+1$ (green line) is approximately constant at $V_{i+1} \approx V_\mathrm{r}$ during this period. Burst onset time difference as function of G is shown in **(c)**; simulations (symbols) of a one-dimensional chain without noise (Eq. (1-5) with $D = 0$) compared to Eq. O (solid line). The dashed green line shows the BOTD approximation via numerical shooting, which is discussed below in Sec. . The inset shows the inverse of the BOTD for the simulation and shooting method for a larger range of G.

the membrane potentials of the bursting and quiescent neurons as $V_{i-1} = \bar{V}_\mathrm{b}$ and

$V_{i+1} = V_\mathrm{r}$, respectively, leading to

$$\tau_\mathrm{V} \frac{dV_i}{dt} = a(V_i - V_\mathrm{r})(V_i - V_\mathrm{c}) + G(V_{i-1} + V_{i+1} - 2V_i) \quad \text{(K)}$$
$$\approx a(V_i - V_\mathrm{r})(V_i - V_\mathrm{c}) + G(\bar{V}_\mathrm{b} - V_i) + G(V_\mathrm{r} - V_i) \quad \text{(L)}$$

This equation can be recast into the form of Eq. A with complex $V_{1,2} = V_\mathrm{m} \pm i\gamma$ in the case of propagating bursts, where

$$V_\mathrm{m} = \frac{V_\mathrm{r} + V_\mathrm{c} + 2G/a}{2}, \qquad \text{and} \quad \text{(M)}$$
$$\gamma = \sqrt{V_\mathrm{r} V_\mathrm{c} + (\bar{V}_\mathrm{b} + V_\mathrm{r})G/a - V_\mathrm{m}^2}. \quad \text{(N)}$$

For the calculation of T_B we can then employ Eq. E, yielding

$$T_\mathrm{B} = -T_\mathrm{ISI} + \frac{\tau_\mathrm{V}}{\gamma a}\left[\arctan\left(\frac{V_\mathrm{peak} - V_\mathrm{m}}{\gamma}\right) - \arctan\left(\frac{V_\mathrm{r} - V_\mathrm{m}}{\gamma}\right)\right]. \quad \text{(O)}$$

A comparison between calculated burst onset times according to Eq. O and T_B obtained from the simulation of a one-dimensional chain is shown in Fig. A(c). The approximation of T_B (Eq. O, solid line) shows reasonable agreement with the simulation results (symbols), in particular for small values of G. This is so, because our averaging argument is most reasonable at weak coupling between the neurons (similar to standard approximations for weakly coupled oscillators [S1]).. As stated in the main text, we approximate the propagation speed of waves passing through a two-dimensional network of neurons by Eq. 9. This corresponds to the assumption that the wave's front is reasonably well approximated by a planar shape, if far enough from its origin. As displayed in Fig. 3(b), the propagation mechanism can then be mimicked by a one-dimensional situation with rescaled distance and coupling strength.

In order to see this, note that neurons of the same color in Fig. 3(b) that are part of a perfectly planar wave front share exactly the same state (V, u). If the voltage of all horizontal neighbors is identical, links between these neurons can be discarded, because the GJ current is zero. We set the first spike time of the bursts of all red neurons as time origin. Now, every single blue neuron feels an excitatory current from two bursting neurons (connected via the links indicated in red/blue). The leak current of this one blue neuron is affected by the two links connecting this neuron to two yellow neurons (indicated by blue/yellow lines in Fig. 3(b)), which are to a good approximation at rest at this instant of time. Doubling the excitatory current and the additional leak current via GJs can be expressed by doubling the GJ conductance parameter G in Eq. O. Last but not least, the propagation of the wave within one burst onset time difference is not in direction of the link, but we have to consider the reduced distance $\sqrt{3/4} \cdot \ell$. Putting everything together, we obtain Eq. 9.

Propagation Speed in the Continuum Limit via Numerical Shooting

To discuss the BOTD in an alternative approach, we would like to calculate the velocity of burst propagation using the one-dimensional version of our system in the continuum limit. Following the literature (e.g. [S2]), we construct a traveling front applying singular perturbation theory and numerical shooting.

To this end, we discard the dynamics of the slower gating variable u, constraining it to its resting value u_r and use the recast expressions introduced above, cf. Eq. K. In the continuum limit, the GJ coupling can be interpreted as a diffusion term, resulting in:

$$\frac{\partial V}{\partial t} = \frac{a}{\tau_\mathrm{V}}(V - V_r)(V - V_c) + \frac{G}{\tau_\mathrm{V}} \frac{\partial^2 V}{\partial x^2} \quad \text{(P)}$$

For similar reaction-diffusion systems, the velocity of the traveling wave has often been determined using the following ansatz: $V(x,t) = V(x - st) = V(\xi)$, so that $\frac{\partial V}{\partial t} = -s\frac{dV}{d\xi}$ and $\frac{\partial^2 V}{\partial x^2} = \frac{d^2 V}{d\xi^2}$. Accordingly, we find

$$\frac{d^2V}{d\xi^2} = -\frac{\tau_V s}{G}\frac{dV}{d\xi} - \frac{a}{G}(V - V_r)(V - V_c). \tag{Q}$$

The parameter s represents the velocity of the traveling wave, such that ξ is a coordinate in a frame moving with velocity s. This second order ODE is transformed into two first-order ODEs, here introducing $W = \frac{dV}{d\xi}$, such that

$$\frac{dV}{d\xi} = W \tag{R}$$

$$\frac{dW}{d\xi} = -\frac{\tau_V s}{G}W - \frac{a}{G}(V - V_r)(V - V_c). \tag{S}$$

Obtaining the front solution $(V,W)^T(\xi)$, and thereby s, requires the definition of boundary conditions. The resulting boundary value problem can be solved by numerical shooting. The idea of this method is to solve a boundary value problem by treating it as an initial value problem (which can simply be evaluated by numerical integration) and change the parameters of the system, until the boundary conditions are fulfilled.

The "left" boundary condition in our case is given by the resting state of the neuron and therefore

$$\lim_{\xi\to-\infty}\begin{pmatrix}V(\xi)\\W(\xi)\end{pmatrix} = \begin{pmatrix}V_r\\0\end{pmatrix}. \tag{T}$$

This fixed point is a saddle and, for the numerical shooting method, the system is initialized in its vicinity on the unstable manifold, cf. Sec. 6.2.1 in [S2]. Next, the parameter s is iteratively changed, until the "right" boundary condition is reasonably well recovered. We assume this right boundary condition to be

$$\begin{pmatrix}V(\xi_{\text{right}})\\W(\xi_{\text{right}})\end{pmatrix} = \begin{pmatrix}V_{\text{peak}}\\0\end{pmatrix}, \tag{U}$$

where ξ_{right} denotes the value of ξ at the right boundary, which is not infinite because it is reached after finite time. Note that this does not represent a fixed point as common in other applications of numerical shooting. In the original discrete and discontinuous model Eq. (1-3), $V = V_{\text{peak}}$ is the effective threshold upon reaching of which the membrane potential is reset to V_{reset}. There is no such reset in the one-dimensional continuum approximation. To mimic this threshold behavior, we choose V_{peak} as the end point of the numerical shooting.

Further, we chose $\lim_{\xi\to\infty} W(\xi) = 0$, which may seem rather arbitrary. There is actually a range of values for the parameter s, that lead to $\lim_{\xi\to\infty} V(\xi) = V_{\text{peak}}$. We observe that there is a lower bound for the wave speed $|s|$, for which the trajectory of the system meets the point $(V,W)^T = (V_{\text{peak}}, 0)^T$. (With our chosen traveling wave ansatz, the values of s are negative, corresponding to waves traveling to the left.) For smaller values of $|s|$, the membrane potential $V = V_{\text{peak}}$ is not met. The BOTDs that correspond to the calculated lower bound $|s|$ are shown in Fig. A(c) as dashed green line. They underestimate the simulated values, especially for smaller values of G, but provide a correct order of magnitude estimation.

The inset of Fig. A(c) shows the magnitude of the wave speed s (green dashed line, obtained via numerical shooting), compared to the inverse of $T_{\text{B}}^{\text{sim}}$ (black dots). For larger GJ conductances, results obtained by these two methods converge and scale as

$G^{1/2}$. This may seem irrelevant for stage I retinal waves in a biological sense, because we are fairly far from the physiologically plausible range for G. However, it is interesting to see this strikingly good agreement and could argue the applicability of numerical shooting methods for studying discrete and discontinuous bursting neuron models, such as ours.

Numerical Simulation Methods

The numerical simulations of our system were performed according to the following Euler-Maruyama integration scheme:

$$\begin{aligned} V_i(t_{k+1}) &= V_i(t_k) + \frac{\Delta t}{\tau_{\mathrm{V}}}\bigg(a(V_i(t_k) - V_{\mathrm{rest}})(V_i(t_k) - V_{\mathrm{crit}}) \\ &\quad - u_i(t_k)\bigg) + \sqrt{2D\Delta t}\xi_{i,k} + RI_i(t_k), \qquad \text{(V)} \\ u_i(t_{k+1}) &= u_i(t_k) + \frac{\Delta t}{\tau_{\mathrm{u}}}\bigg(bV_i(t_k) - u_i(t_k)\bigg), \qquad \text{(W)} \end{aligned}$$

where $\xi_{i,k}$ are independent Gaussian random numbers with unit variance [S3]. The simulation results shown in Fig. 1, Fig. 2, and Fig. 3 are deterministic, i.e. $D = 0$. The wave nucleation rates shown in Fig. 4 correspond to an average of three independent simulations for each set of parameters G, N, and D, where every single simulation was run until a fixed number of spikes was generated $(7500 \cdot N)$, such that a single simulation returned roughly 200 inter-wave intervals; for cases with very low nucleation rates $(< 10^{-6})$, fewer inter-wave intervals were simulated due to a hard-coded time limit. All simulations were performed at discrete times with a step of $\Delta t = 0.1$ msec. To test the stability of the Euler-Maruyama integration scheme for our network model for $D > 0$, we compared simulations for the nucleation rate, cf. Fig. 4, with different integration time steps at the set of parameters: $G = 0.4$, $N = 100$, $D = 1/18$. We found that reducing the integration time step by a factor 10 had no significant impact on the result of the nucleation rate.

In Fig. 4, we find a finite size effect that vanishes for larger system sizes. The boundary conditions introduce a measurable effect on the spontaneous nucleation rate of retinal waves for smaller system sizes. This effect is due to correlations of distant neighbors in the system. To gain a better understanding of the magnitude of correlation on voltage fluctuations, we simulated a one-dimensional chain of neurons. Because we are interested in the sub-threshold voltage fluctuations we simplified the neuron model to a version that does not generate spikes, i.e. with a linearized deterministic part of the dynamics (GJ and noise current unchanged) at the stable fixed point:

$$\begin{aligned} \tau\dot{V} &= f(V) + RI \\ &\approx f(V_{\mathrm{r}}) + \frac{\partial f}{\partial V}|_{V=V_{\mathrm{r}}}(V - V_{\mathrm{r}}) + RI \\ &= a(V_{\mathrm{r}} - V_{\mathrm{c}})(V - V_{\mathrm{r}}) + RI \qquad \text{(X)} \end{aligned}$$

Simulation results for the Pearson correlation coefficient of the membrane potential as function of distance for a ring of 15 coupled neurons are illustrated in Fig. B(a). With increasing coupling strength, there is a non-zero correlation between the voltages, even for neurons as far as 4 space units apart. This leads to a measurable increase of the overall membrane fluctuations of a ring with chain length up to 5 neurons for $G = 0.5$, cf. Fig. B(b). For larger chain lengths, the periodicity has no effect on the voltage fluctuations, which is in agreement of the saturation observed for ΔU in Fig. 4.

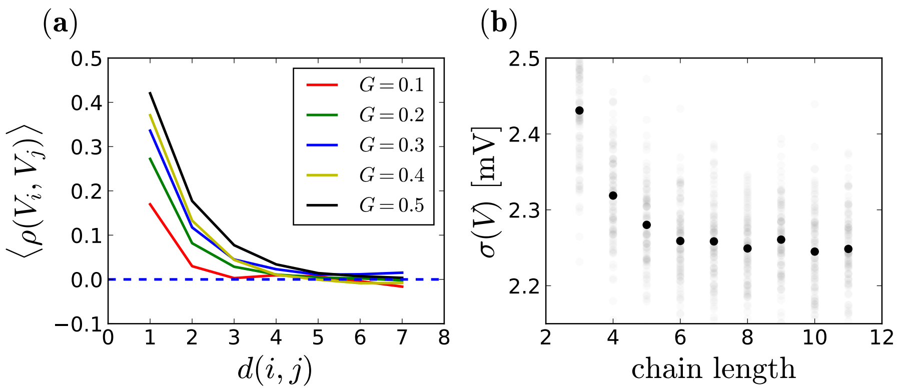

Figure B. GJ coupling leads to correlations that affect the noise intensity. Pearson correlation coefficient of the voltage as function of the distance between neurons in **(a)**. Standard deviation of the membrane potential as function of the chain length for $G = 0.5$ and $D = 0.05$ in **(b)**. Each point corresponds to an average of 100 simulations of 1000 seconds for a single neuron (the result of single simulations is illustrated by transparent symbols).

The results for the wave speed shown in Fig. 5 were obtained by averaging the properties of roughly the 20 first waves (by limiting the total number of evaluated spikes to $600 \cdot N$) from one large scale simulation. The results for the inter-wave intervals were obtained by averaging over all recorded inter-wave intervals of one simulation for every set of parameter. Simulations were run for 5000 seconds with a break criteria at approximately 300 waves ($7500 \cdot N$ spikes). Panel (b) of Fig. 5 shows the first 2000 seconds of the population activity for three exemplary noise intensities. Separate simulations were performed to estimate the amplitude of the subthreshold voltage fluctuations; to this end, we used the membrane potential of 20 neurons for 20 seconds, in which no wave was observed.

References

S1. Ermentrout GB, Kopell N. Frequency plateaus in a chain of weakly coupled oscillators, I. SIAM journal on Mathematical Analysis. 1984;15(2):215–237.

S2. Ermentrout GB, Terman DH. Mathematical foundations of neuroscience. vol. 35. Springer Science & Business Media; 2010.

S3. Risken H. The Fokker-Planck equation: methods of solution and applications. Springer; 1984.
